# Qualitative and Quantitative Comparison of the Proteome of Erythroid Cells Differentiated from Human iPSCs and Adult Erythroid Cells by Multiplex TMT Labelling and NanoLC-MS/MS

**DOI:** 10.1371/journal.pone.0100874

**Published:** 2014-07-14

**Authors:** Kongtana Trakarnsanga, Marieangela C. Wilson, Rebecca E. Griffiths, Ashley M. Toye, Lee Carpenter, Kate J. Heesom, Steve F. Parsons, David J. Anstee, Jan Frayne

**Affiliations:** 1 School of Biochemistry, University of Bristol, Bristol, United Kingdom; 2 Department of Biochemistry, Faculty of Medicine Siriraj Hospital, Mahidol University, Bangkok, Thailand; 3 Bristol Institute for Transfusion Sciences, National Health Service Blood and Transplant (NHSBT), Filton, Bristol, United Kingdom; 4 Blood Research Laboratory, National Health Service Blood and Transplant, John Radcliffe Hospital, Oxford, United Kingdom; Indian Institute of Toxicology Research, India

## Abstract

Induced pluripotent stem cells (iPSC) are an attractive progenitor source for the generation of *in vitro* blood products. However, before iPSC-derived erythroid cells can be considered for therapeutic use their similarity to adult erythroid cells must be confirmed. We have analysed the proteome of erythroid cells differentiated from the iPSC fibroblast derived line (C19) and showed they express hallmark RBC proteins, including all those of the ankyrin and 4.1R complex. We next compared the proteome of erythroid cells differentiated from three iPSC lines (C19, OCE1, OPM2) with that of adult and cord blood progenitors. Of the 1989 proteins quantified <3% differed in level by 2-fold or more between the different iPSC-derived erythroid cells. When compared to adult cells, 11% of proteins differed in level by 2-fold or more, falling to 1.9% if a 5-fold threshold was imposed to accommodate slight inter-cell line erythropoietic developmental variation. Notably, the level of >30 hallmark erythroid proteins was consistent between the iPSC lines and adult cells. In addition, a sub-population (10–15%) of iPSC erythroid cells in each of the iPSC lines completed enucleation. Aberrant expression of some cytoskeleton proteins may contribute to the failure of the majority of the cells to enucleate since we detected some alterations in cytoskeletal protein abundance. In conclusion, the proteome of erythroid cells differentiated from iPSC lines is very similar to that of normal adult erythroid cells, but further work to improve the induction of erythroid cells in existing iPSC lines or to generate novel erythroid cell lines is required before iPSC-derived red cells can be considered suitable for transfusion therapy.

## Introduction

The generation of human red blood cells (RBCs) *in vitro* for transfusion purposes is a major goal of health services globally. In recent years advances in the development of systems for the generation of erythrocytes *in vitro* have progressed rapidly using progenitor cells isolated from a variety of different stem cell sources. Of these, induced pluropotent stem cells (iPSC) have great potential to provide an inexhaustible source of progenitors for the generation of large numbers of RBCs, and to facilitate the innovative development of allogeneic and rare blood group products for transfusion purposes.

Induced pluripotent stem cells were first established in 2006 by Takahashi and Yamanaka [1] who used retrovirus to transduce 24 pluripotency associated genes into mouse fibroblasts, identifying four genes, Oct-4, SOX-2, C-myc and Klf-4, required to mediate reprogramming. The cells are similar to embryonic pluripotent stem cells (ESCs) in their morphology, pluripotency marker expression, self-renewal property and ability to differentiate into the three primary germ layers both *in vivo* and *in vitro* [2], [3], [4], [5], [6], [7]. However, they do not have the ethical barriers of ESCs [4]. Moreover, a retrospective study by Peyrard *et al*. (2011) [8] has shown that RBCs generated from only 15 human iPSC lines could cover the needs of patients of Western Caucasians.

A number of groups [2], [9], [10] have reported successful differentiation of iPSCs down the erythroid lineage using a variety of culture systems, generating orthochromatic erythroblasts and reticulocytes (up to 10%), although Kobari *et al* (2012) [11] have reported up to 26% enucleation for erythroid cells differentiated from iPSCs from an individual with SCD. The differentiated cells in all reports expressed fetal and embryonic globins, indicating reprogramming of the globin locus from the original parental cell.

Such reports highlight the potential for generating RBCs *in vitro* from iPSC. However, to date erythroid differentiation has been confirmed only by morphological analysis and expression of a very limited number of RBC markers, including glycophorin A (CD235a) and transferrin receptor (CD71) [9], [10]. Functionally, Kobari *et al* (2012) [11] have shown that the reticulocytes generated from iPSC exhibit a similar oxygen binding capacity to cord blood RBCs, which contain predominantly fetal hemoglobin. A more detailed characterization and a comprehensive analysis of the protein expression profile of erythroid cells generated *in vitro* from iPSCs, in comparison to that of normal adult erythroid cells, is required to determine how similar these cells actually are to normal erythroid cells and to identify key deficiencies in iPSC-derived erythroid cells accounting for reduced enucleation efficiency and failure of globin switching. To achieve this we used mass spectrometry to firstly define the proteome of erythroid cells differentiated from the iPSC line C19, demonstrating that these cells express hallmark RBC proteins, including all those of the ankyrin and 4.1R complex, and undergo erythroid specific developmental events. We next took a comparative proteomic approach, utilizing multiplex Tandem Mass Tag (TMT) labeling to compare the proteome of erythroid cells differentiated from three iPSC lines (C19, OCE1, OPM2) with that of adult and cord blood progenitors. Of the 1989 proteins quantified only 1.9% differed in level by 5-fold or more between the iPSC and adult erythroid cells. Notably, the level of >30 hallmark erythroid proteins was consistent between these cells. In addition, a sub-population (10–15%) of iPSC erythroid cells in each of the iPSC lines completed enucleation. We did however detect some alterations in cytoskeletal protein abundance, which may contribute to the failure of the majority of the cells to enucleate. In conclusion, the proteome of erythroid cells differentiated from iPSC lines is very similar to that of normal adult erythroid cells, but further work is required to elucidate the nature of the subtle changes and thus render them suitable for use as a transfusion therapy.

## Methods

### Ethics Statement

LRS cones and cord blood units were obtained from healthy donors with written informed consent for research use in accordance with the Declaration of Helsinki and approved by local Research Ethics Committees (Southmead Research Ethics Committee reference 08/H0102/26 and Bristol Research Ethics Committee reference 12/SW/0199). For the generating OCE1 and OPM2 iPSC lines cord blood and adult peripheral blood was collected anonymously, with informed written consent, and ethics approved by the Oxford and Berkshire Research Ethics Committee and with Institutional R&D approval (REC number 10/H505/34).

### Expansion and hematopoietic differentiation of human iPSC lines

The human iPSC line C19 (passage 20–27) was created, maintained and expanded as described in Carpenter et al (2011) [12]. iPSC lines OCE1 and OPM2 (both passage 12–16) were generated using OriP episomal plasmids containing Oct-4, SOX-2, KLF-4, C-Myc, Nanog and Lin28b to reprogramme adult blood mononuclear cells and cord blood erythroblasts respectively. Hematopoietic differentiation was achieved by co-culture of iPSC with OP9 mouse stromal cells (a gift to LC from Dr. I. Slukvin, University of Wisconsin, Madison, WI; originally from Dr. Nakano, Kyoto University, Japan) in αMEM medium containing 100 µM MTG (Sigma-Aldrich), 100 U/ml penicillin/streptomycin (Sigma-Aldrich) and 10% (v/v) defined FCS (Hyclone Laboratories).

### CD34^+^ isolation and erythropoietic differentiation

CD34^+^ cells were harvested from day 7 iPSC, OP9 co-cultures by dispersal with collagenase type IV (1 mg/ml; Invitrogen) for 20 minutes, followed by trypsin (0.05%; PAA Laboratories) for 15 minutes. The cell suspension was passed through a 40-µm cell strainer (BD Biosciences), centrifuged at 400 g for 5 minutes and the cell pellet re-suspended in cold MACs buffer, prior to CD34^+^ cell isolation. Adult and Cord Blood CD34^+^ cells were isolated and pooled from 16 LRS cones and 4 cord blood units retrospectively. All CD34^+^ cells were isolated as described in Griffiths et al 2012 [13]. For erythropoietic differentiation CD34^+^ cells were incubated in the three-stage culture system described by Griffiths et al 2011 [13].

### Characterisation of erythroid differentiation from CD34^+^ cells

Erythroid differentiation of CD34^+^ cells was confirmed by morphological analysis with May-Grunwald Giemsa reagent, confocal microscopy [13] and western blot with antibodies to α, β and γ-globins (Santa Cruz), adducin α (Santa Cruz), Duffy (Biorad Laboratories), Tubulin (Roche), catenin α (CTNNA1; Invitrogen), Glycophorin A, Rh (BRIC69), protein 4.2 (BRIC272), Glut1, Band 3 (BRIC170), CD44 (BRIC235) (all IBGRL [14], [15], [16]), and Alexa Fluor 635 phalloidin conjugated with actin (Invitrogen).

### Preparation of samples for mass spectrometry and data aquisition

Harvested cells were lysed with RIPA buffer (25 mM Tris pH 7.6, 150 mM NaCl, 1% NP40, 1% sodium deoxycholate, 0.1% SDS, Roche complete protease inhibitor) and the proteins (50 µg) resolved on a 12% acrylamide gel. The gel lane was cut into 10 sections, to decrease protein complexity and increase sensitivity during analysis, and each section subjected to tryptic digest. Peptides were analysed by nanoLC-MS/MS using a Thermo LTQ-Orbitrap Velos mass spectrometer. For multiplexed comparative and quantitative proteomics 100 µg of each cell lysate was digested with trypsin and labelled with Tandem Mass Tag (TMT) reagents according to the manufacturer's protocol (Thermo Fisher Scientific). After labeling, samples were combined in equal amounts, and 50 µg of pooled sample fractionated by strong cation exchange using an Ettan LC system (GE Healthcare) prior to analysis by nanoLC-MS/MS. The raw data files were processed and quantified using Proteome Discoverer software v1.2 (Thermo Scientific) and searched against the UniProt/SwissProt Human database release version 57.3 (20326 entries) using the SEQUEST (Ver. 28 Rev. 13) algorithm. Peptide precursor mass tolerance was set at 10 ppm, and MS/MS tolerance was set at 0.8 Da. Search criteria included oxidation of methionine (+15.9949) as a variable modification and carbamidomethylation of cysteine (+57.0214) and the addition of isobaric mass tags (+229.163) to peptide N-termini and lysine as fixed modifications. Searches were performed with full tryptic digestion and a maximum of 1 missed cleavage was allowed. The reverse database search option was enabled and all peptide data was filtered to satisfy false discovery rate (FDR) of 5%. The Proteome Discoverer software generates a reverse “decoy” database from the same protein database and any peptides passing the initial filtering parameters that were derived from this decoy database are defined as false positive identifications. The minimum cross-correlation factor (Xcorr) filter was readjusted for each individual charge state separately to optimally meet the predetermined target FDR of 5% based on the number of random false positive matches from the reverse decoy database. Thus each data set has its own passing parameters. Quantitation was performed using a peak integration window tolerance of 0.0075 Da with the integration method set as the most confident centroid. Protein ratios represent the median of the raw measured peptide ratios for each protein.

Each protein included in our study was identified from at least 2 peptides with high/medium confidence. Proteins recorded as uncharacterized by the software were returned with a gene ID.

## Results

### Erythroid differentiation of C19 iPSC

**Figure 1:**
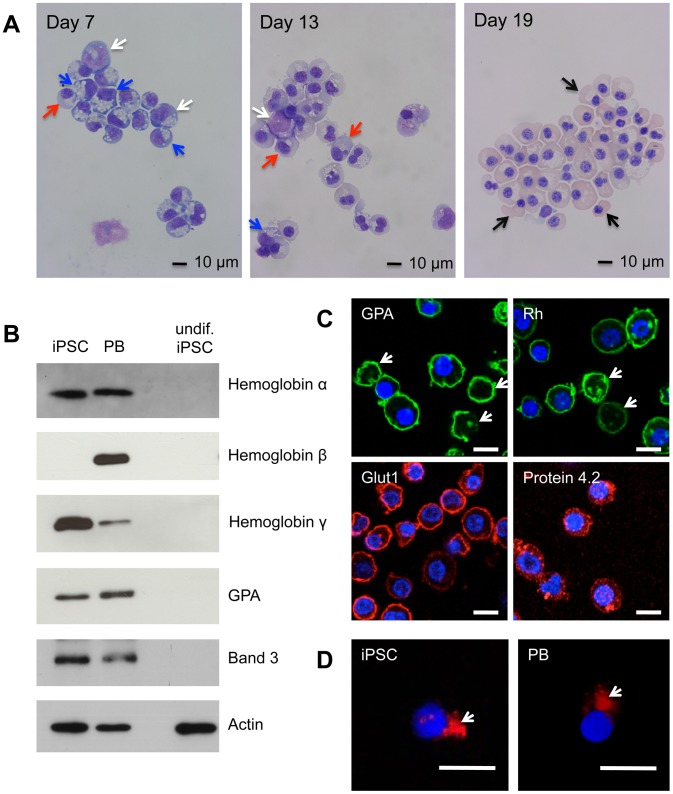
Erythroid differentiation of C19 iPSC CD34^+^ cells. C19 and adult peripheral blood [PB] CD34^+^ cells were incubated for up to 21 days in our three-stage erythroid culture system. (A) Morphological analysis of cells stained with May-Grundwal Giemsa reagent on day 7, 13 and 19 in culture. Arrows, white proerythroblasts, blue basophillic erythroblasts, red polychromatic erythroblasts, black orthochromatic erythroblasts. (B) Western blot of iPSC and PB erythroid cells at day 19 in culture, and undifferentiated [undif] iPSCs, probed with antibodies to α-,β- and γ-globin, GPA and Band 3. Antibodies to actin were used as a protein loading control. Numbers on left are size markers. (C) iPSC erythroid cells at day 19 in culture probed with antibodies to GPA, Rh, GLUT1 and Protein 4.2, followed by compatible secondary antibodies with Alexa Fluor 488 (green) or Alexa Fluor 635 phalloidin (red). Arrows indicate reticulocytes. (**D**) Erythroid cells differentiated from C19 iPSC and PB progenitors were incubated with Alexa Fluor 635 phalloidin conjugated actin antibody (red). Arrows indicate contractile actin rings. Nuclear DNA was stained with blue-fluorescent DAPI. Images were obtained using a Leica SP5 confocal microscope. Scale bars 10 µm.

We first analysed erythroid cells differentiated from C19 iPSC. The C19 iPSC line was generated from human dermal fibroblasts [12]. CD34+ cells isolated following hematopoietic differentiation were incubated for 21 days in our erythroid culture system [13]. Morphological analysis showed differentiation and maturation of the CD34^+^ cells down the erythroid lineage (Figure 1A). This was confirmed by expression of globin isoforms, Band 3, GPA, Rh, Glut1 and protein 4.2 (Figure 1B and C). These proteins were not expressed in undifferentiated iPSCs. Up to 15% (10–15%) reticulocytes were detected, as determined by GPA staining and confocal microscopy (Figure 1C). Those cells observed undergoing enucleation had morphologically normal contractile actin rings, as compared to enucleating adult cells when stained for F-actin (Figure 1D). Cultures routinely contained 5–10% contamination with other cell types including neutrophils and macrophage-like cells, as well as a small number of binucleated cells. We validated the appearance of cellular organelles in the nucleated erythroid cells using antibodies to well characterized organelle marker proteins, in comparison with erythroid cells generated *in vitro* from adult peripheral blood (PB) CD34^+^ cells (both at day 12 in culture (Figure S1). The appearance and localization of all organelle markers tested was similar between erythroid cells derived from C19 and adult cells, no obvious abnormalities were detected.

### The proteome of erythroid cells generated from C19 iPSC

We analysed the proteome of erythroid cells at day 21 of culture by nanoLC-MS/MS. 129,106 peptides were identified, belonging to 3,313 unique proteins (Table S1). The data was first analysed using WebGestalt GSAT V2 software, with 2,633 of the proteins classified by cellular component, molecular function and biological process (Figure S2A, B and C).

Interrogation of the day 21 C19 erythroid cell proteome data revealed no residual expression of the pluripotency proteins Oct-4, SOX-2 and KLF-4 used to generate the iPSC, or of SSEA-3, SSEA-4, Tra-1-81 and Nanog, markers of pluripotent stem cells that were used to confirm success of the original reprogramming [12].

Our western blot analysis (Figure 1B) showed expression of γ-(fetal) but not β-(adult) globin in C19 erythroid cells. The proteome data showed, in fact all globin subunits are expressed by these cells, although the levels of β-globin are very low (Table S2).

#### Expression of hallmark red cell antigens

**Figure 2:**
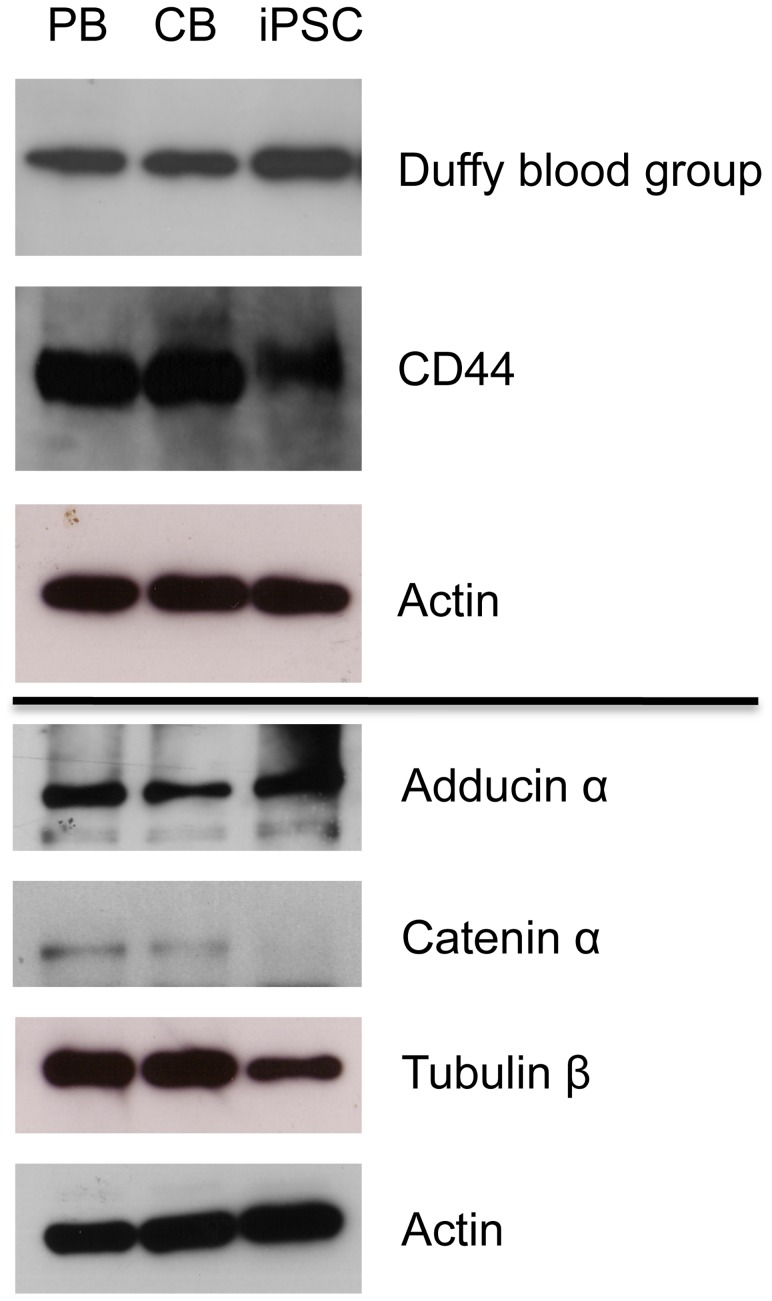
Levels of selected erythroid proteins in erythroid cells differentiated from iPSC, PB and CB. Western blot of day 19 adult [PB], cord blood [CB] and C19 iPSC erythroid cell lysate (30 µg) probed with antibodies to Duffy and CD44, and day 8 lysate probed with antibodies to adducing α, catenin α (CTNNA1) and Tubulin β. Antibodies to actin were used as a protein loading control at both time points.

Certain membrane and cytoskeleton proteins are essential for the structure and function of RBCs. Many of the key membrane proteins associate to form two multi-protein complexes, the band 3-ankyrin macro-complex17 [17], and the 4.1R junctional complex, linking the red cell membrane to the underlying cytoskeleton [17], [18], [19], [20]. The latter complex proposed predominantly from studies in the mouse [21]. Importantly, all proteins in these complexes, except the Duffy blood group, were detected in our day 21 C19 erythroid cell proteome analysis mass spec data (Table 1). Duffy is a large, glycosylated protein with few trypsin sites resulting in a large peptide that is not compatible with mass spec identification. Duffy expression in the C19 erythroid cells was therefore confirmed by western blot (Figure 2). Other notable RBC membrane proteins expressed by the C19 erythroid cells were aquaporin 1 and CD44. CD44 was reported in the mass spectrometry data, but there was no unique peptide. Therefore expression was confirmed by western blot (Figure 2).

**Table 1:** Hallmark red cell proteins in erythroid cells differentiated from C19 iPSCs analysed by nanoLC-MS/MS.

Accession	Coverage	PSMs	Peptides	Score	Description
Q02094	7.09	9	2	30.71	Ammonium transporter Rh type A
Q02161	11.99	11	3	28.59	Blood group Rh(D)
P02730	45.23	572	30	2056.13	Band 3 anion transport protein
B8Q185	36.75	36	3	169.79	Glycophorin A
P04921	21.09	11	2	57.71	Glycophorin-C
Q14773	45.39	17	9	51.69	Intercellular adhesion molecule 4
Q4KKX0	71.08	130	37	432.18	EPB42 protein
P16157	62.63	487	85	1690.93	Ankyrin-1
Q13813	5.95	10	8	24.32	Spectrin alpha chain, brain
P02549	62.79	635	141	2227.79	Spectrin alpha chain, erythrocyte
Q01082	4.95	53	12	142.26	Spectrin beta chain, brain 1
P11277	66.45	604	124	2042.01	Spectrin beta chain, erythrocyte
E9PB22	5.45	3	2	6.71	CD47
B7Z844	9.98	8	3	17.71	GLUT1 (SLC2A14)
Q08495	42.47	55	13	167.73	Dematin
B4DM17	6.96	5	3	13.68	Beta-adducin
P11171	47.45	155	33	519.18	Protein 4.1
P28289	54.32	37	14	113.74	Tropomodulin-1
Q9NYL9	16.76	8	4	28.63	Tropomodulin-3
Q5VU58	48.39	92	15	313.49	Tropomyosin 3
Q5VU66	41.13	102	14	338.34	Tropomyosin 3
P67936	34.68	35	10	111.93	Tropomyosin alpha-4 chain
P68032	63.13	817	24	2394.31	Actin, alpha cardiac muscle 1
P62736	63.13	657	24	1884.66	Actin, aortic smooth muscle
P60709	83.73	2372	32	8781.23	Actin, cytoplasmic 1
P51811	6.76	6	3	16.27	Membrane transport protein XK
P23276	25.27	42	12	157.84	Kell blood group glycoprotein
Q00013	74.25	172	26	620.17	55 kDa erythrocyte membrane protein

Coverage; the percentage of the protein sequence covered by identified peptides. PSMs; the total number of identified peptide sequences for the protein. Peptides; the number of peptide sequences identified. Score; the total score of the protein which is the sum of all peptide Xcorr values above the specified score threshold. Note, peptide number is not a measure of protein abundance as different peptides, in particular some of those from integral membrane proteins, are difficult to detect by MS.

### Qualitative and quantitative comparison of the proteome of erythroid cells generated from C19, OCE1 and OPM2 iPSC lines with that of adult and cord blood erythroid cells

We next wanted to determine whether the level of proteins in the C19 erythroid cells was equivalent to that of normal adult erythroid cells using multiplex tandem mass tag (TMT) peptide labeling together with nanoLC-MS/MS to simultaneously and directly compare multiple proteomes. To explore whether the proteome of erythroid cells differentiated from the C19 iPSC line differs from that of cells differentiated from other iPSC lines, we also included two additional iPSC lines, OPM2 and OCE1. These were generated using OriP episomal plasmids containing Oct-4, SOX-2, KLF-4, c-Myc, Nanog and Lin 28 to reprogramme adult blood mononuclear cells and cord blood erythroblasts respectively. Finally, as the C19 erythroid cells express predominantly fetal and embryonic-type globins, and may therefore be closer developmentally to fetal cells, we also included erythroid cells differentiated from cord blood progenitors.

Hematopoietic and erythroid differentiation of C19, OCE1 and OPM2 iPSC was performed. All differentiated down the erythroid lineage synchronously, as determined by morphological analysis (Figure S3). On day 19 the majority of cells were orthochromatic normoblasts with between 10–15% reticulocytes, as identified by confocal microscopy and expression of GPA (data not shown).

For the TMT analysis we selected erythroid cells differentiated from the three iPSC lines, PB and CB CD34^+^ cells at day 8 in culture for optimal synchronicity (Table S3); after this time synchronicity of iPSC, with the PB and CB cultures decreases, making direct comparative proteome analysis inaccurate. Furthermore, we have previously found that the majority of proteins in adult erythroid cells are detected by day 8 (J.F., unpublished data). Harvested cells were lysed, the proteins subjected to trypsin digestion and resultant peptides labeled with isobaric tags and analysed by nanoLC-MS/MS. 2,084 proteins were identified of which 1,989 were quantified (Table S4). All proteins were detected in erythroid cells from all progenitor sources.

#### Differential expression of proteins between C19, OCE1 and OPM2 erythroid cells

We first compared the proteomes of erythroid cells differentiated from the three iPSC lines. The total number of quantified proteins varying in level by 2 fold or more were 3%, 4.5% and 0.8% between OCE1 and C19, OCE1 and OPM2 and OPM2 and C19 respectively. Moreover, the majority of proteins varied in level by only 2–4 fold, with only 3, 3 and 2 proteins above this level, respectively. Two of these, lysozyme and myeloperoxidase, are expressed by the small number of contaminating neutrophils, the abundance of which varied between the cultures (see Table S3) The other proteins that varied in abundance between the iPSC lines were γ-globin (10 and 6 fold higher in OPM2 and OCE1 compared to C19), BASP1 (4.5 fold higher in OPM2 compared to OCE1), Filamin (4.4 fold higher in OCE1 compared to C19) and SH3KBP1 (4.1 fold higher in OCE1 compared to C19).

#### Differential expression of proteins between adult and iPSC-derived erythroid cells

We compared the proteomes of erythroid cells generated *in vitro* from the three iPSC clones with adult erythroid cells. Ninety-one, 176 and 96 proteins were at least 2-fold more abundant in PB compared to C19, OCE1 and OPM2 erythroid cells respectively. Of these, 60 proteins were common between the different iPSC erythroid cells (Figure S4A). Conversely, 109, 86 and 103 proteins were more abundant in C19, OCE1 and OPM2 compared to PB erythroid cells, respectively. Of these, 71 proteins were common between the different iPSC erythroid cells (Figure S4B). However, as there was a slight difference in differentiation rate between the iPSC and adult cultures (Table S3) we refined our analysis to include only proteins that differed in level by 5 fold or more between samples. This value was selected as the average number of proerythroblasts was 4.3 fold higher in the PB compared to the iPSC cultures, and the average number of polychromatic and orthochromatic cells was 6.8 fold higher in the iPSC cultures compared to PB. At this threshold 11, 18 and 13 proteins were more abundant in PB compared to C19, OCE1 and OPM2 cells respectively, of which 8 were common between the iPS erythroid cells (Figure S4C; Table S5A). 26, 19 and 25 more abundant in C19, OCE1 and OPM2 compared to PB erythroid cells respectively, of which 12 were common between the iPSC erythroid cells (Figure S4D; Table S5B).

**Figure 3:**
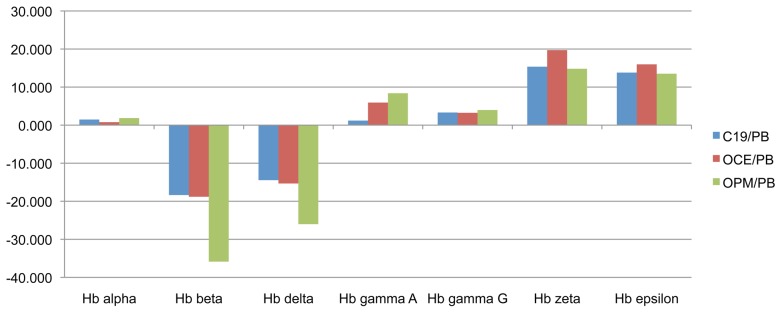
Difference in the level of globin subunits between erythroid cells differentiated in vitro from adult peripheral blood (PB) CD34^+^ cells, and from C19, OCE1 and OPM2 CD34^+^ cells. PB, C19, OCE1 and OPM2 erythroid cells at day 8 in culture were lysed, proteins subjected to trypsin digest and resultant peptides labeled with isobaric tags for nanoLC-MS/MS based quantitation and comparison. Y-axis represents the fold difference in protein level between erythroid cells differentiated from each iPSC line and adult PB progenitors.

C19 erythroid cells express all globin isoforms (Figure S2). To obtain more quantitative information we examined globin subunits in the comparative proteome data. The level of α-globin was equivalent between the PB and iPS erythroid cells, whereas β–globin was more abundant in the PB, and both isoforms of γ-globin in the iPSC erythroid cells. ζζ-and ε-globin were more abundant, and at a very similar level, in the iPSC erythroid cells (Figure 3).

**Figure 4:**
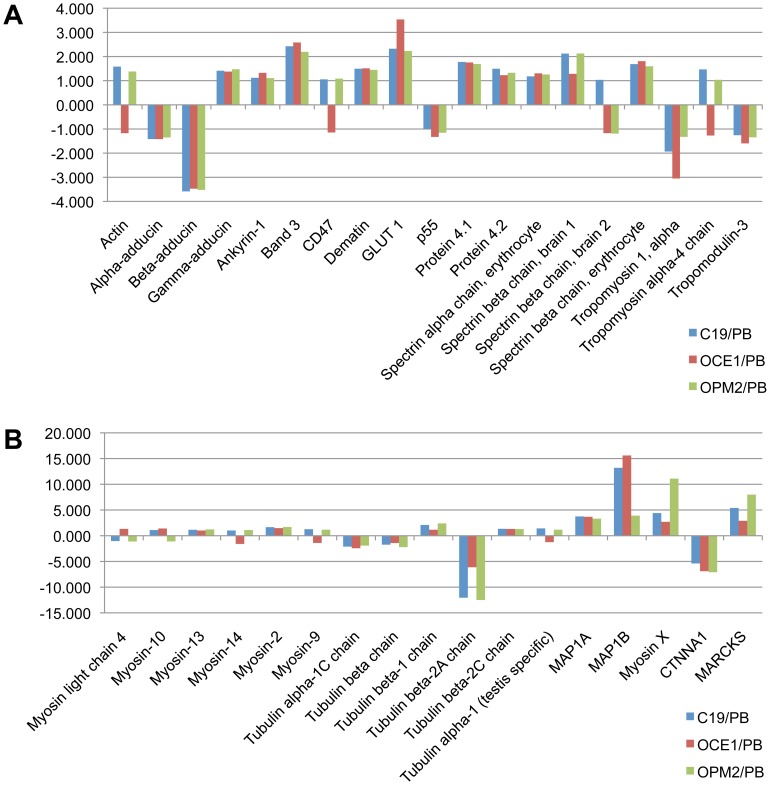
Difference in the level of RBC hallmark and cytoskeleton proteins between erythroid cells differentiated in vitro from adult peripheral blood (PB) CD34^+^ cells, and from C19, OCE1 and OPM2 CD34^+^ cells. Erythroid cells differentiated from PB progenitors, C19, OCE1 and OPM2 iPSCs at day 8 in culture were lysed, proteins subjected to trypsin digest and resultant peptides labeled with isobaric tags for nanoLC-MS/MS based quantitation and comparison. Y-axis represents the fold difference in protein level between erythroid cells differentiated from each iPSC line and adult PB progenitors.

We next examined the data to determine whether hallmark RBC proteins differ in level between adult and iPS erythroid cells. As can be seen in Figure 4A the level of 19 proteins that were quantified showed very little, if any variation between the PB and iPS erythroid cells. Furthermore, the levels of 6 isoforms of myosin and 5 of tubulin were equivalent between the PB and iPS erythroid cells (Figure 4B). However, interestingly, the cytoskeleton or cytoskeleton interacting proteins Tubulin β-2A, MAP1B, CTNNA1 (catenin α) and MARCKS did show a notable difference in abundance (Figure 4B).

To confirm the proteomic data we compared the level of selected proteins in PB and iPSC erythroid cells by western blot (Figures 1B and 2), and obtained equivalent data. Levels of α-globin, GPA, Band 3, adducin α and Duffy were consistent between the cells. Levels of β-globin were higher in PB andγ-globin higher in iPSCs. CTNNA1 was at a higher level in PB than iPSCs. To confirm the proteome data for Tubulin β-2A we used a Tubulin β antibody, which showed a lower level in the iPSC compared to PB cells.

Other notable features in the proteome data were the levels of nuclear RNPs (35 quantified) and splicing factors (15 quantified), which were consistent between the iPSC and PB erythroid cells (Table S4). An important example of a tightly regulated RNA processing event during erythroid differentiation is the splicing of cytoskeleton protein 4.1. Protein 4.1 undergoes alternative splicing with the expression of exon 16, critical for spectrin-actin binding, only in late erythroid cells [22], [23]. When we examined the peptide data for protein 4.1 in our day 21 C19 erythroid cells we found that exon 16 derived peptides were present. In addition, the level of hnRNPA1, which represses exon 16 inclusion in earlier cells, was consistent between all three iPSC and adult erythroid cells on day 8 in culture. This confirms that iPSC erythroid cells are undergoing expected erythroid specific developmental events.

Conversely, the level of histones (16 quantified) was higher in the iPSC compared to the PB erythroid cells (Table S6). CD36 (platelet glycoprotein IV) was more abundant in adult than iPSC erythroid cells (5.4, 7.9 and 5.7 fold higher than in C19, OCE1 and OPM2 cells, respectively).

**Figure 5:**
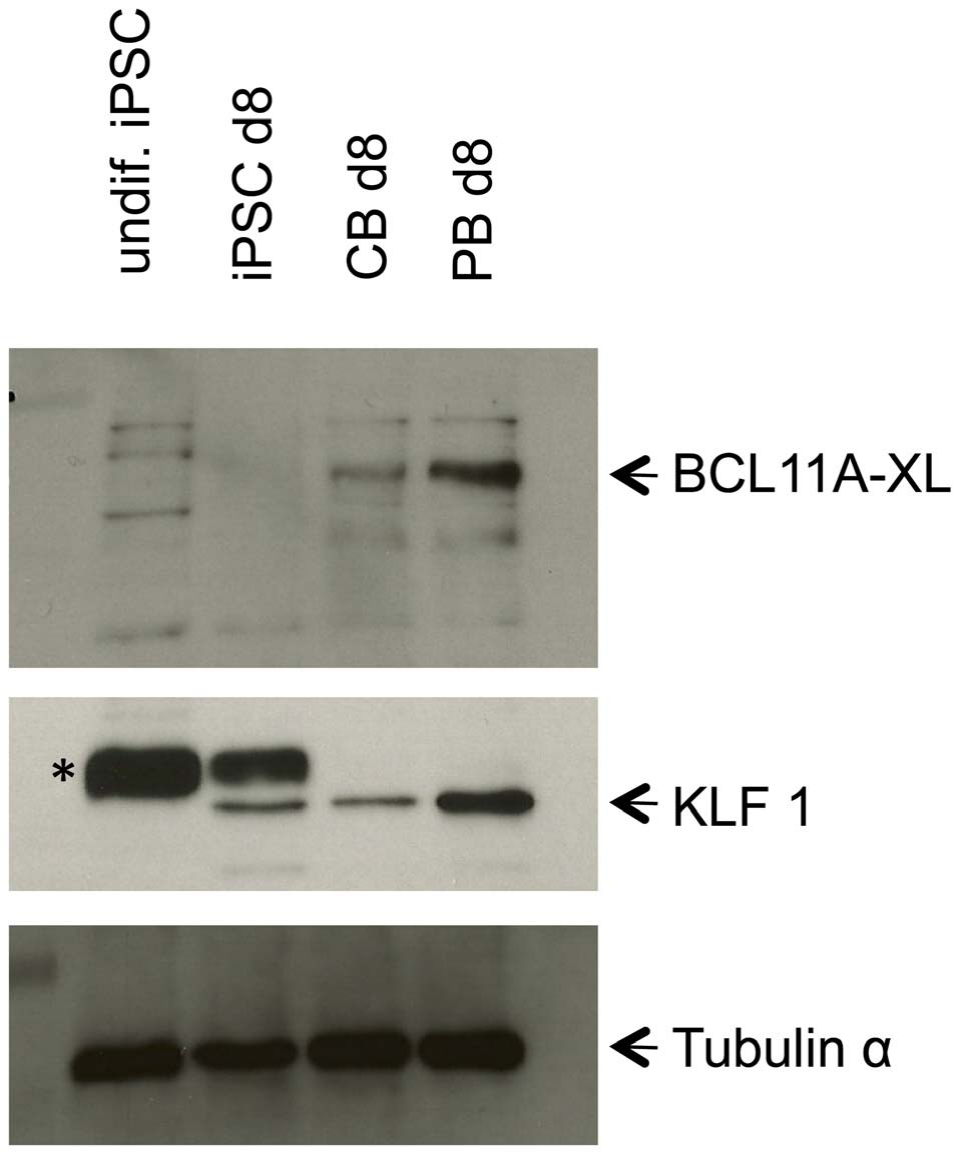
Expression of BCL11A and KLF1 in erythroid cells differentiated *in vitro* from C19 iPSC, cord blood (CB) and adult peripheral blood (PB) CD34^+^ cells. Western blot of total protein from undifferentiated iPSCs (undif iPSC), and erythroid cells differentiated from iPSCs, CB and PB CD34^+^ cells at day 8 in culture probed with BCL11A antibody, stripped and re-probed with KLF1 antibody and stripped and re-probed with an antibody to tubulin as a protein loading control. * indicates non-specific band detected by antibody.

Although transcription factors were detected they were clearly underrepresented, for example BCL11A and KLF1 were absent. As these two transcription factors are essential for adult globin expression [24], [25], [26], and the latter for regulating the expression of many erythroid genes [27], [28], we compared their expression in PB, CB and iPSC C19 erythroid cells at day 8 in culture by western blot (Figure 5). BCL11A was detected in both PB and, at a lower level in CB but was not detected in the C19 cells. KLF1 was detected in PB, CB and C19 erythroid cells. The level was similar between CB and C19 cells, but lower than PB. Neither transcription factor was detected in the undifferentiated C19 iPSCs.

#### Differential expression of proteins between CB and IPSC-derived erythroid cells

Finally, we compared the proteome of CB and iPSC erythroid cells (Table S4). Eight to ten percent of proteins were differentially expressed 2 fold or more between CB and iPSC erythroid cells (10%, 8%, 10% for C19, OCE1 and OPM2 respectively). When a 5-fold threshold was applied to account for slight differences in the differentiation rate (Table S3), these values dropped to 1.8%, 1.3% and 1.5% respectively.

**Figure 6:**
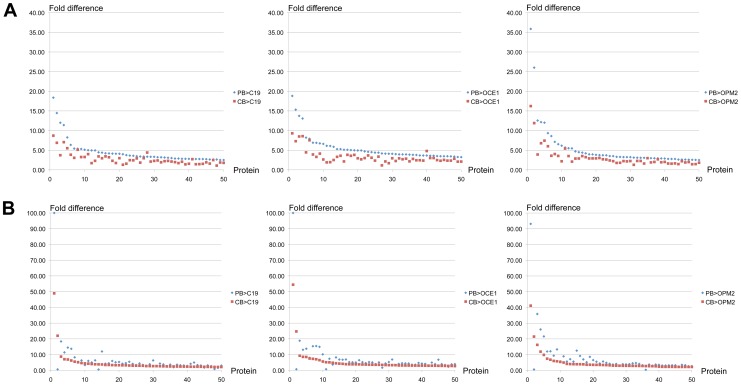
Difference in the level of proteins between adult (PB) and iPSC erythroid cells, compared to that between cord blood (CB) and iPSC erythroid cells. PB, C19, OCE1 and OPM2 erythroid cells at day 8 in culture were lysed, proteins subjected to trypsin digest and resultant peptides labeled with isobaric tags for nanoLC-MS/MS based quantitation and comparison. The levels of 50 proteins with the greatest differential expression between (A) PB and C19, OCE1 or OPM2 erythroid cells and (B) CB and C19, OCE1 or OPM2 erythroid cells were all compared between PB and the 3 iPSC lines and CB and the 3 iPSC lines. Each point on the x-axis represents a unique protein. Y-axis represents the fold difference in protein level between erythroid cells differentiated from PB or CB and the iPSC line.

Hence, the number of differentially expressed proteins between PB and iPSC erythroid cells is similar to that between CB and iPSC-derived erythroid cells. However, when the magnitude of difference in protein levels is examined the iPSC-derived erythroid cell proteomes were in fact closer to that of the CB cells. To determine this we used the 50 proteins with the largest increase in level in PB, and the 50 in CB, and compared both to the iPSC-derived erythroid cells. The PB values were used for the baseline. For almost all proteins there was a greater difference in level between PB and iPSC, than between CB and iPSC-derived erythroid cells (Figure 6).

## Discussion

The aim of our study was to determine how similar erythroid cells differentiated from iPSC are to adult erythroid cells generated *in vitro*, and whether there are any gross differences which could impact on their potential for use as therapeutic source of red cells. Reassuringly, our initial proteome analysis of orthochromatic erythroid cells differentiated from C19 iPSCs revealed no major differences regarding expression of hallmark RBC proteins, particularly all known components of the 4.1R and ankyrin membrane complexes. Of these >30 were identified in the proteome data from earlier erythroid cells, the level of which was consistent between all three iPSC (C19, OCE1 or OPM2) and adult erythroid cells. Further interrogation of the comparative proteome data showed that the level of 50 proteins involved in RNA processing was also consistent between the iPSC and adult erythroid cells.

There was however differential expression of some of the 1989 quantified proteins between the iPSC and adult cells, 11% and 1.9% for a 2 fold and 5 fold threshold respectively. These values were similar to those between CB and iPSC erythroid cells, 9.3% and 1.5% respectively. Some of the differences will be due to intrinsic variability between individuals. However, our data suggest that the proteome of the iPSC erythroid cells is more aligned to that of CB (fetal type) cells. An obvious feature of fetal erythroid cells is the expression of γ-globin. The iPSC erythroid cells expressed γ- but little β-globin, likely due, at least in part, to the low level of KLF1 and absence of BCL11A in these cells, both of which are known to be required for the developmental switch from fetal to adult globin expression [26], [27]. However, the iPSC-derived erythroid cells expressed a higher level of embryonic globins than CB cells. We therefore investigated the expression of proteins reported to be unique to embryonic erythroid cells [29] to see if the iPSC-derived cells exhibited a more embryonic phenotype. We detected SLC1A5, SLC3A2, SLC39A8, FACE-1 and SYPL1 in our proteome data (Table S5), but the level of these proteins was consistent between the iPSC, adult and cord blood erythroid cells. Hence, importantly, our iPSC derived erythroid cells do not appear to be more embryonic in phenotype. Moreover, expression of embryonic globin is not restricted to embryonic cells as increased expression has been reported in adult erythroid cells with reduced levels of wild-type KLF1 and compound heterozygotes for mutant KLF1 [30], [31].

The concept of epigenetic memory in cells differentiated from iPSCs is still under debate [32]. We found that the proteome of erythroid cells differentiated from three types of iPSCs extremely similar, with less than 3% of proteins differentially expressed. Literature and database searches of all differentially expressed proteins revealed no data that could relate them to their original donor cell type. For example, Protein S100-A4 (FSP1) is highly expressed by fibroblasts, and is used as a sensitive marker for these cells [33], but was actually detected at a slightly higher level in the adult than the C19 (fibroblast iPSC line) erythroid cells. Some of the identified differences may again be due to intrinsic variability, as the donor cells were derived from different individuals.

The enucleation rate in our iPSC erythroid cultures, and the cultures of other groups [9], [10], [11] is low compared to rates achievable (up to 95%) for adult peripheral blood CD34^+^ erythroid cultures. The enucleation process involves the coordinated and timely remodelling of cytoskeleton components [13], [14], [34], [35], [36], [37]. The level of many of these proteins was invariant between the iPSC and adult erythroid cells. However, a number of proteins did differ in abundance, a feature that may result in disruption of the remodelling process and inhibit downstream enucleation. Proteins with a notable difference in level included Tubulin β-2A. Altering the distribution, or inhibiting the polymerization, of Tubulin 2A is known to inhibit enucleation in mouse erythroblasts [36]. Catenin (cadherin-associated protein) alpha 1 (CTNNA1), may link E-cadherin and actin filaments and regulate actin filament assembly [38], [39]. Microtubule-associated protein 1B, which along with MAP1A, stabilizes microtubules and interacts with actin and signaling molecules [40]. Myristoylated alanine-rich C-kinase substrate (MARCKS), an actin filament crosslinking protein [41]. Additional studies are required to determine whether aberrant levels of such proteins perturb enucleation in iPSC. Furthermore, the significance of higher levels of histone proteins in iPSC compared to both adult and cord erythroid cells merits investigation. Notwithstanding, we observed that the small proportion of cells that do enucleate do so in a manner comparable to adult erythroid cells (Figure 1D) raising the possibility that iPSC lines are heterogenous, with a minority population following the complete erythropoietic pathway. Forward programming of cells and optimization of culture conditions are being investigated to increase enucleation rates.

In conclusion, to our knowledge this is the first study to describe a comprehensive characterization, and comparison of the proteome of erythroid cells derived *in vitro* from iPSC with adult and cord erythroid cells. Our data show that with respect to protein expression iPSC-derived erythroid cells have an extremely similar phenotype to adult erythroid cells. Further work is required to elucidate the nature of the subtle changes that must be induced *in vitro* in order to convert iPSC-derived erythroid cells into an adult phenotype and render them suitable for use as a transfusion therapy. Such cells also present as a novel model system for studying the process of erythropoiesis and defects in the system that result in disease.

## Supporting Information

Figure S1 **Localisation of cytoplasmic organelles in erythroid cells differentiated from C19 iPSCs and adult peripheral blood CD34^+^ cells.** C19 iPSC and adult peripheral blood [PB] CD34^+^ cells were incubated in our three-stage erythroid culture system. Cells were harvested on day 12 and incubated with antibodies to CD63 (BD Biosciences), Lamp1 (Abcam), Giantin (Covance), Ubiquitin (Abcam), Clathrin (BD Biosciences), LC3 (MBLI) and Calreticulin (Abcam) followed by compatible secondary antibodies with Alexa Fluor 488 (green) or Alexa Fluor 635 phalloidin (red). Nuclear DNA was stained with blue-fluorescent DAPI. Images were obtained using a Leica SP5 confocal microscope with Leica software (scale bar 10 µm).
(TIF)

Figure S2 **Subcellular location (A), metabolic processes (B) and biological functions (C) of proteins expressed by C19 iPSC erythroid cells at day 19 in culture.** Erythroid cells were lysed and proteins resolved by 1D gel electrophoresis before in gel trypsin digest and analysis by nanoLC-MS/MS. 2,633 proteins were identified from at least 2 peptides and analysed using WebGestalt GSAT V2.
(TIF)

Figure S3 **Morphological analysis of erythroid cells differentiated in vitro from C19, OCE1 and OPM2 CD34^+^ cells. C19, OCE1 and OPM2 CD34^+^ cells were incubated for up to 19 days in our three-stage erythroid culture system, with cells on day 8 and 19 stained with May-Grundwal Giemsa reagent.** Scale bar 10 µm. Arrows, white proerythroblasts, blue basophillic erythroblasts, red polychromatic erythroblasts, black orthochromatic erythroblasts.
(TIF)

Figure S4 **Venn diagrams showing the number of proteins that differed in level between erythroid cells differentiated from adult peripheral blood (PB) CD34^+^ cells, compared to erythroid cells differentiated from C19, OCE1 and OPM2 CD34^+^ cells.** PB, C19, OCE1 and OPM2 erythroid cells at day 8 in culture were lysed, proteins subjected to trypsin digest and resultant peptides labeled with isobaric tags for nanoLC-MS/MS based quantitation and comparison. (A) Number of proteins 2-fold or more abundant in PB compared to C19, OCE1 and OPM2 erythroid cells. (B) Number of proteins 2-fold or more abundant in C19, OCE1 and OPM2 compared to PB erythroid cells. (C) Number of proteins 5-fold or more abundant in PB compared to C19, OCE1 and OPM2 erythroid cells (D) Number of proteins 5-fold or more abundant in C19, OCE1 and OPM2 compared to PB erythroid cells.
(TIF)

Table S1 **Proteins identified in erythroid cells differentiated from C19 iPSCs at day 21 in culture.** Only proteins identified by 2 or more peptide were included. Coverage; the percentage of the protein sequence covered by identified peptides. PSMs; the total number of identified peptide sequences for the protein, including those redundantly identified. Peptides; the number of peptide sequences identified for that protein. Score; the total score of the protein which is the sum of all peptide Xcorr values above the specified score threshold. The score threshold is calculated as followed: *0.8 + peptide charge x peptide relevance factor* where ‘peptide relevance factor’ is an advanced parameter of the SEQUEST node in the ‘Protein scoring option’ category with a default value of 0.4.
(XLS)

Table S2 **Globin subunits expressed by erythroid cells differentiated from C19 iPSCs at day 21 in culture.** All proteins were identified by MS/MS from 2 or more peptides, including at least one unique peptide. Peptides were assigned to δ-globin, however as no unique peptide was identified for this isoform it is not included in the Table. For explanation of column labels see legend for Table S1.
(DOCX)

Table S3 **Percentage of different cell types in adult blood, cord blood, C19, OCE1 and OPM2 erythroid cultures, on day 8.** Cells were stained with May-Grundwal Giemsa and 200 cells were counted from each sample.
(DOCX)

Table S4 **Comparison of the level of proteins between erythroid cells differentiated from adult peripheral blood (PB), cord blood (CB), C19, OCE1 and OPM2 CD34^+^ cells, at day 8 in culture.** For explanation of column labels see legend for Table S1.
(XLS)

Table S5 **Proteins more abundant by 5 fold or more in (A) erythroid cells differentiated from adult peripheral blood compared to C19, OCE1 and OPM2 CD34^+^ cells, (B) erythroid cells differentiated from C19, OCE1 and OPM2 compared to adult peripheral blood CD34^+^ cells.** Numbers in italics are below the 5-fold threshold. For explanation of column labels see legend for Table S1.
(DOCX)

Table S6 **Comparison of the level of histone proteins between erythroid cells differentiated from adult peripheral blood (PB) compared to C19, OCE1 and OPM2 CD34^+^ cells, and between cord blood (CB) compared to C19, OCE1 and OPM2 CD34^+^ cells, at day 8 in culture.**
(DOCX)
