# Supplementary figures and images for: Qualitative and Quantitative Comparison of the Proteome of Erythroid Cells Differentiated from Human iPSCs and Adult Erythroid Cells by Multiplex TMT Labelling and NanoLC-MS/MS
Source: PLoS One. 2014 Jul 14;9(7):e100874. doi: 10.1371/journal.pone.0100874 (PMC4096399; doi:10.1371/journal.pone.0100874)

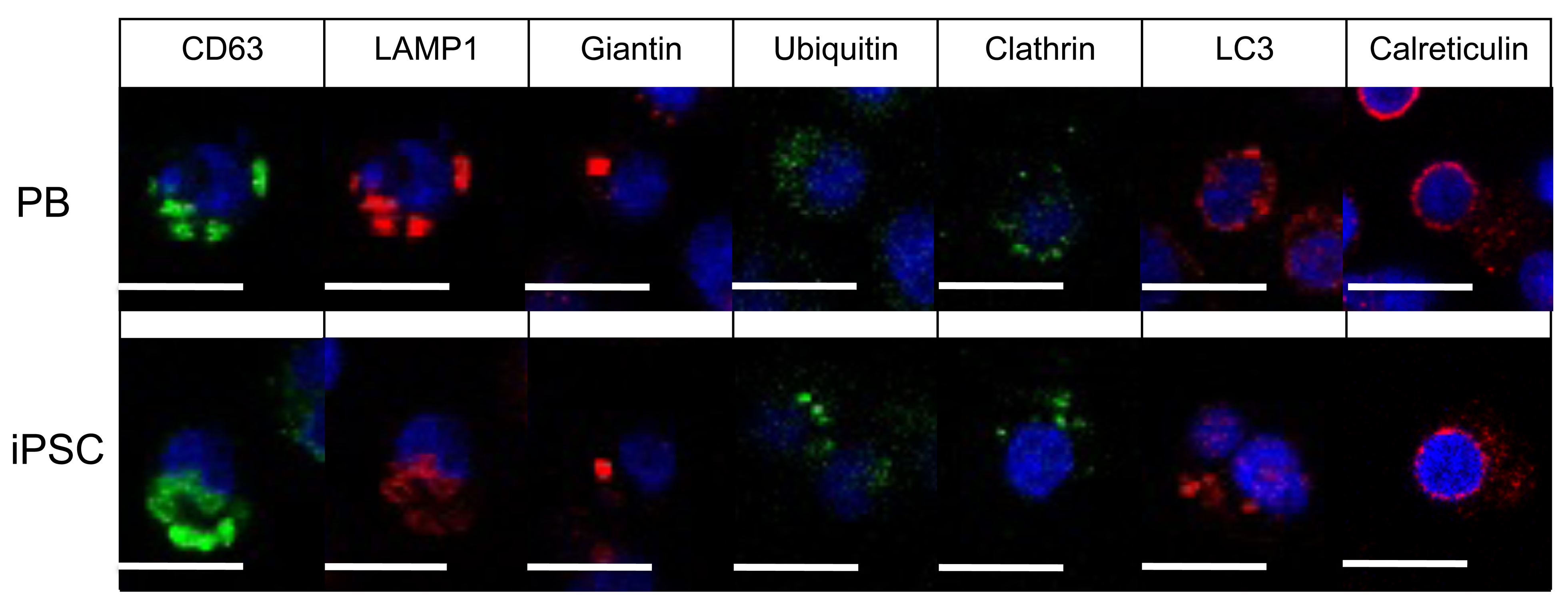

Supplement: Figure S1 — Localisation of cytoplasmic organelles in erythroid cells differentiated from C19 iPSCs and adult peripheral blood CD34+ cells. C19 iPSC and adult peripheral blood [PB] CD34+ cells were incubated in our three-stage erythroid culture system. Cells were harvested on day 12 and incubated with antibodies to CD63 (BD Biosciences), Lamp1 (Abcam), Giantin (Covance), Ubiquitin (Abcam), Clathrin (BD Biosciences), LC3 (MBLI) and Calreticulin (Abcam) followed by compatible secondary antibodies with Alexa Fluor 488 (green) or Alexa Fluor 635 phalloidin (red). Nuclear DNA was stained with blue-fluorescent DAPI. Images were obtained using a Leica SP5 confocal microscope with Leica software (scale bar 10 µm). (TIF) [file pone.0100874.s001.tif]

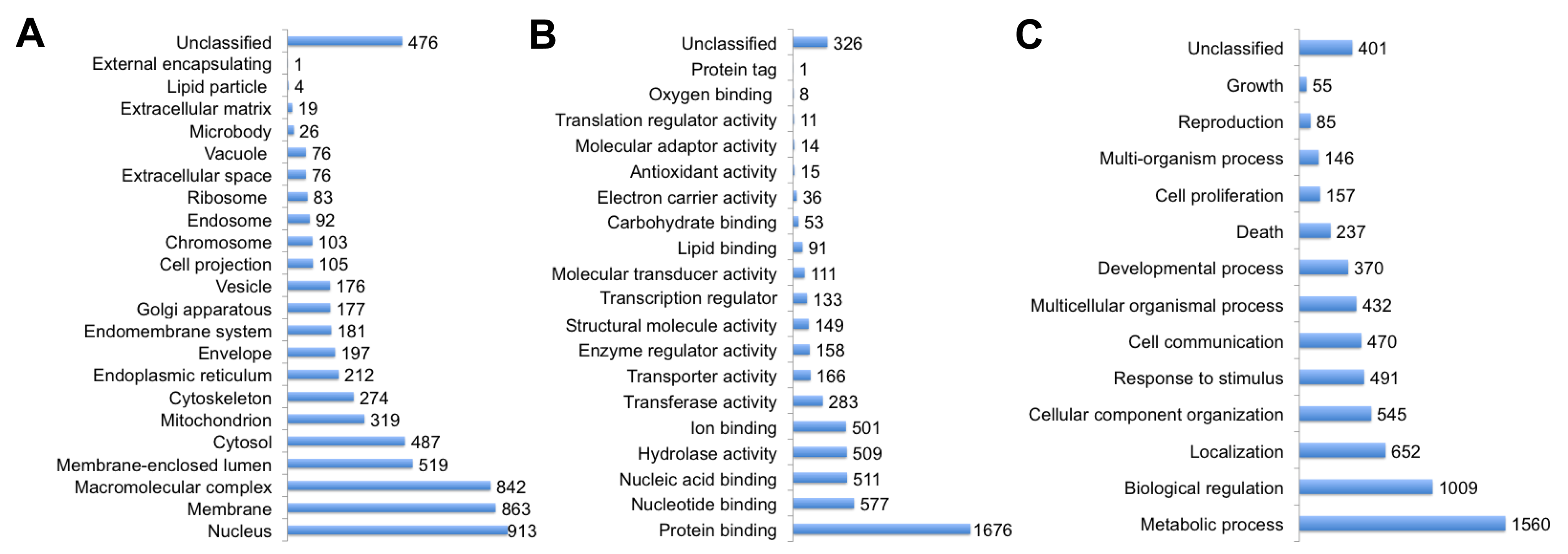

Supplement: Figure S2 — Subcellular location (A), metabolic processes (B) and biological functions (C) of proteins expressed by C19 iPSC erythroid cells at day 19 in culture. Erythroid cells were lysed and proteins resolved by 1D gel electrophoresis before in gel trypsin digest and analysis by nanoLC-MS/MS. 2,633 proteins were identified from at least 2 peptides and analysed using WebGestalt GSAT V2. (TIF) [file pone.0100874.s002.tif]

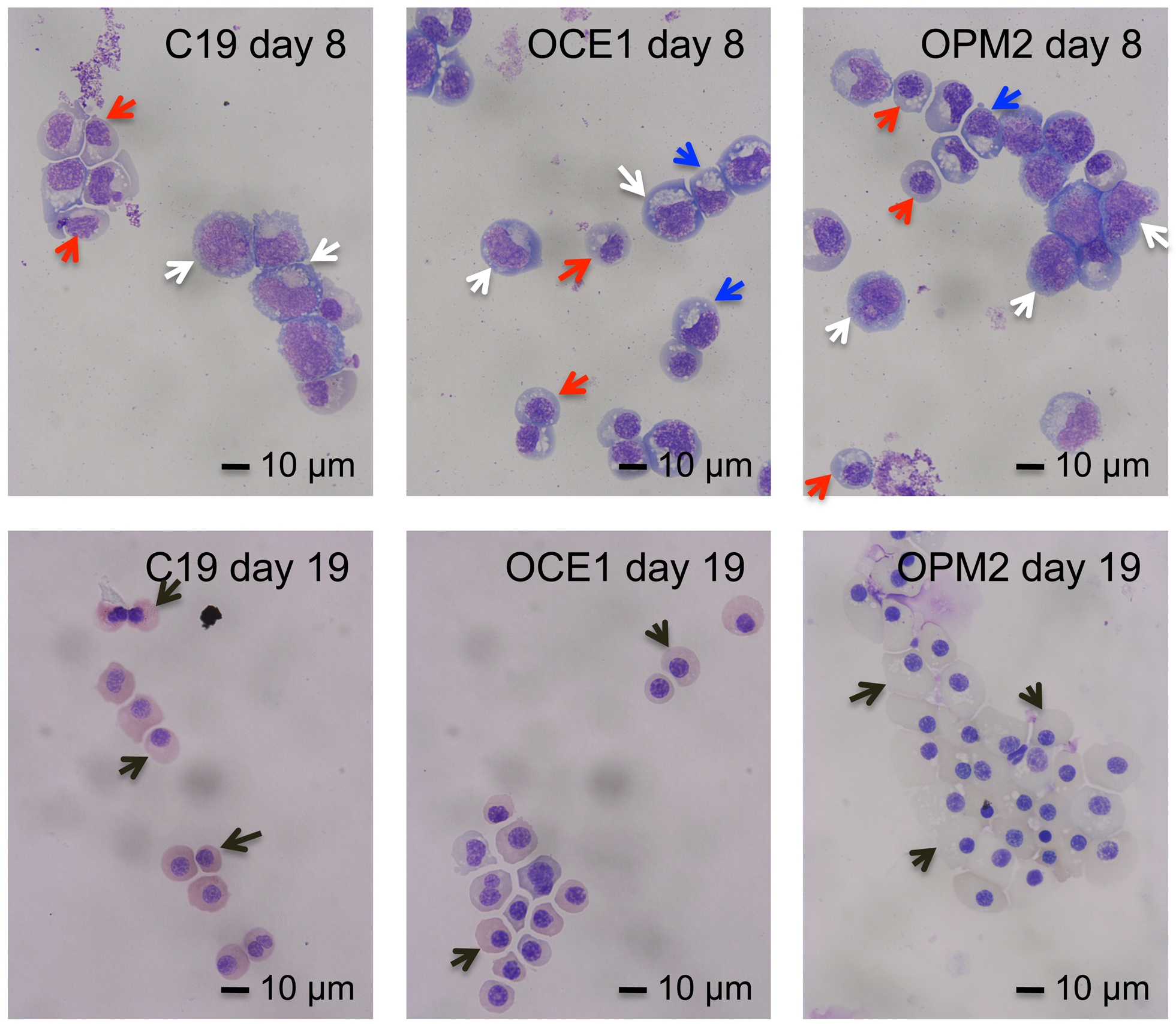

Supplement: Figure S3 — Morphological analysis of erythroid cells differentiated in vitro from C19, OCE1 and OPM2 CD34+ cells. C19, OCE1 and OPM2 CD34+ cells were incubated for up to 19 days in our three-stage erythroid culture system, with cells on day 8 and 19 stained with May-Grundwal Giemsa reagent. Scale bar 10 µm. Arrows, white proerythroblasts, blue basophillic erythroblasts, red polychromatic erythroblasts, black orthochromatic erythroblasts. (TIF) [file pone.0100874.s003.tif]

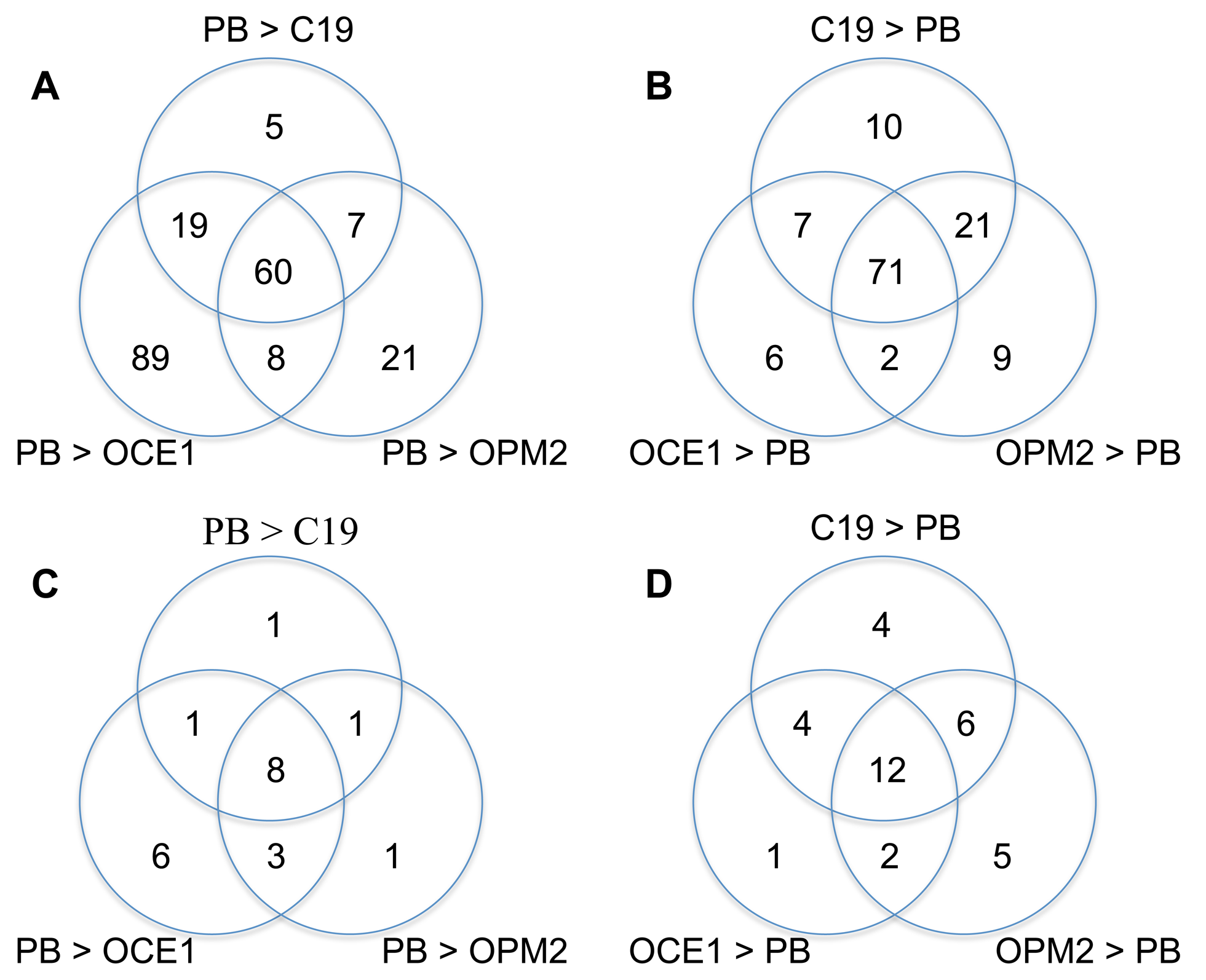

Supplement: Figure S4 — Venn diagrams showing the number of proteins that differed in level between erythroid cells differentiated from adult peripheral blood (PB) CD34+ cells, compared to erythroid cells differentiated from C19, OCE1 and OPM2 CD34+ cells. PB, C19, OCE1 and OPM2 erythroid cells at day 8 in culture were lysed, proteins subjected to trypsin digest and resultant peptides labeled with isobaric tags for nanoLC-MS/MS based quantitation and comparison. (A) Number of proteins 2-fold or more abundant in PB compared to C19, OCE1 and OPM2 erythroid cells. (B) Number of proteins 2-fold or more abundant in C19, OCE1 and OPM2 compared to PB erythroid cells. (C) Number of proteins 5-fold or more abundant in PB compared to C19, OCE1 and OPM2 erythroid cells (D) Number of proteins 5-fold or more abundant in C19, OCE1 and OPM2 compared to PB erythroid cells. (TIF) [file pone.0100874.s004.tif]
