# Supplementary material for: Qualitative and Quantitative Comparison of the Proteome of Erythroid Cells Differentiated from Human iPSCs and Adult Erythroid Cells by Multiplex TMT Labelling and NanoLC-MS/MS
Source: PLoS One. 2014 Jul 14;9(7):e100874. doi: 10.1371/journal.pone.0100874 (PMC4096399; doi:10.1371/journal.pone.0100874)
Supplement: Table S2 — Globin subunits expressed by erythroid cells differentiated from C19 iPSCs at day 21 in culture. All proteins were identified by MS/MS from 2 or more peptides, including at least one unique peptide. Peptides were assigned to δ-globin, however as no unique peptide was identified for this isoform it is not included in the Table. For explanation of column labels see legend for Table S1. (DOCX) [file pone.0100874.s006.docx]

**Table S2. Globin subunits in erythroid cells differentiated from C19 iPSCs analysed by nanoLC-MS/MS.**

| **Accession** | **Coverage** | **PSMs** | **Peptides** | **Score** | **Description** |
| --- | --- | --- | --- | --- | --- |
| P69905 | 100 | 1194 | 16 | 4265.43 | Hemoglobin subunit alpha |
| P68871 | 26.53 | 411 | 3 | 985.82 | Hemoglobin subunit beta |
| P02100 | 97.28 | 4721 | 23 | 15016.96 | Hemoglobin subunit epsilon |
| P69891 | 91.16 | 2117 | 21 | 6932.23 | Hemoglobin subunit gamma-1 |
| P69892 | 89.80 | 2117 | 20 | 6963.91 | Hemoglobin subunit gamma-2 |
| P09105 | 18.31 | 7 | 2 | 19.61 | Hemoglobin subunit theta-1 |
| P02008 | 94.37 | 1809 | 20 | 6053.87 | Hemoglobin subunit zeta |
