# Supplementary material for: Qualitative and Quantitative Comparison of the Proteome of Erythroid Cells Differentiated from Human iPSCs and Adult Erythroid Cells by Multiplex TMT Labelling and NanoLC-MS/MS
Source: PLoS One. 2014 Jul 14;9(7):e100874. doi: 10.1371/journal.pone.0100874 (PMC4096399; doi:10.1371/journal.pone.0100874)
Supplement: Table S3 — Percentage of different cell types in adult blood, cord blood, C19, OCE1 and OPM2 erythroid cultures, on day 8. Cells were stained with May-Grundwal Giemsa and 200 cells were counted from each sample. (DOCX) [file pone.0100874.s007.docx]

**Table S3. Percentage of different cell types in adult blood, cord blood, C19, OCE1 and OPM2 erythroid cultures, on day 8.**

|  | **Adult** | **Cord** | **C19** | **OCE1** | **OPM2** |
| --- | --- | --- | --- | --- | --- |
| Proerythroblasts | 73 | 67 | 16 | 15 | 20 |
| Basophillic erythroblasts | 18 | 23 | 12 | 22 | 8 |
| Polychromatic erythroblasts | 8 | 10 | 40 | 42 | 37 |
| Orthochromatic erythroblasts | 1 | 0 | 12 | 13 | 18 |
| Cells with 2 nuclei | 0 | 0 | 9 | 2 | 3 |
| Neutrophils | 0 | 0 | 7 | 6 | 10 |
| Macrophage-like cells | 0 | 0 | 4 | 3 | 4 |
