# Supplementary material for: Qualitative and Quantitative Comparison of the Proteome of Erythroid Cells Differentiated from Human iPSCs and Adult Erythroid Cells by Multiplex TMT Labelling and NanoLC-MS/MS
Source: PLoS One. 2014 Jul 14;9(7):e100874. doi: 10.1371/journal.pone.0100874 (PMC4096399; doi:10.1371/journal.pone.0100874)
Supplement: Table S5 — Proteins more abundant by 5 fold or more in (A) erythroid cells differentiated from adult peripheral blood compared to C19, OCE1 and OPM2 CD34+ cells, (B) erythroid cells differentiated from C19, OCE1 and OPM2 compared to adult peripheral blood CD34+ cells. Numbers in italics are below the 5-fold threshold. For explanation of column labels see legend for Table S1. (DOCX) [file pone.0100874.s009.docx]

**Table S5. Proteins that were more abundant in erythroid cells differentiated from (A) adult PB and (B) iPSCs C19, OCE1 and OPM2 at day 8 in erythroid culture.**

**A**

| **Accession** | **Coverage** | **PSMs** | **Peptides** | **Score** | **PB/C19** | **PB/OCE1** | **PB/OPM2** | **Description** |
| --- | --- | --- | --- | --- | --- | --- | --- | --- |
| P68871 | 70.07 | 133 | 9 | 661.809 | 18.371 | 18.810 | 35.862 | Hemoglobin subunit beta |
| P02042 | 57.14 | 94 | 8 | 479.638 | 14.456 | 15.320 | 26.013 | Hemoglobin subunit delta |
| Q13885 | 32.36 | 159 | 13 | 615.031 | 11.983 | 6.120 | 12.555 | Tubulin beta-2A chain |
| Q96DG6 | 21.22 | 7 | 6 | 30.904 | 11.403 | 13.730 | 12.014 | Carboxymethylenebutenolidase homolog |
| P06703 | 34.44 | 19 | 4 | 99.412 | 8.254 | 13.067 | 9.348 | Protein S100-A6 |
| P17931 | 10.8 | 3 | 3 | 15.678 | 6.351 | 8.205 | 6.538 | Galectin-3 |
| Q8N1C0 | 5.78 | 3 | 3 | 43.200 | 5.418 | 6.913 | 7.120 | CTNNA1 protein |
| D9IX68 | 5.21 | 3 | 2 | 26.934 | 5.381 | 7.916 | 5.692 | Platelet glycoprotein IV variant |
| F5H6F4 | 6.51 | 4 | 4 | 27.848 | 5.315 | 6.742 | *4.691* | Acetyl-coenzyme A synthetase 2-like |
| F5GX07 | 12.24 | 2 | 2 | 22.215 | 5.125 | *4.701* | 5.502 | Oligoribonuclease |
| E9PDE4 | 18.08 | 13 | 11 | 101.273 | 5.008 | *4.586* | *4.390* | DNA-directed RNA polymerase I subunit RPA34 |
| P35527 | 19.42 | 24 | 11 | 126.198 | *4.957* | 6.599 | 6.152 | Keratin, type I cytoskeletal 9 |
| B7Z1N6 | 19.69 | 30 | 7 | 134.728 | *4.951* | 6.876 | *3.314* | Fructose-bisphosphate aldolase |
| P62879 | 11.76 | 10 | 4 | 50.207 | *4.137* | *3.876* | 12.149 | Guanine nucleotide-binding protein G(I)/G(S)/G(T) subunit beta-2 |
| P04264 | 29.35 | 28 | 19 | 161.902 | *4.106* | 5.859 | 5.470 | Keratin, type II cytoskeletal 1 |
| Q05315 | 26.76 | 19 | 6 | 124.753 | *3.979* | 6.079 | *1.848* | Eosinophil lysophospholipase |
| E5RH81 | 21.59 | 5 | 4 | 28.185 | *3.753* | 5.120 | 8.598 | Carbonic anhydrase 1 |
| P16150 | 5.5 | 5 | 2 | 20.841 | *3.627* | 5.278 | *2.903* | Leukosialin |
| Q6UYC3 | 39.9 | 62 | 26 | 306.255 | *3.144* | 5.041 | *3.711* | Lamin A/C |
| P26447 | 36.63 | 26 | 5 | 119.174 | *2.315* | 5.105 | *2.332* | Protein S100-A4 |
| P09382 | 40.74 | 13 | 6 | 58.924 | *2.116* | 5.281 | *1.895* | Galectin-1 |

**B**

| **Accession** | **Coverage** | **PSMs** | **Peptides** | **Score** | **C19/PB** | **OCE1/PB** | **OPM2/PB** | **Description** |
| --- | --- | --- | --- | --- | --- | --- | --- | --- |
| Q16777 | 37.21 | 79 | 5 | 477.416 | 18.435 | 20.578 | 14.334 | Histone H2A type 2-C |
| P02652 | 21 | 15 | 4 | 72.807 | 16.857 | 16.561 | 8.254 | Apolipoprotein A-II |
| P02008 | 80.28 | 299 | 10 | 1410.928 | 15.356 | 19.705 | 14.810 | Hemoglobin subunit zeta |
| P02100 | 81.63 | 595 | 12 | 2964.229 | 13.808 | 15.971 | 13.512 | Hemoglobin subunit epsilon |
| P46821 | 2.55 | 8 | 6 | 68.109 | 13.222 | 15.550 | *3.915* | Microtubule-associated protein 1B |
| P05109 | 52.69 | 10 | 6 | 59.309 | 11.739 | 7.472 | 17.529 | Protein S100-A8 |
| A8MW06 | 45.45 | 44 | 4 | 180.394 | 10.582 | *3.249* | 5.961 | Thymosin beta-4-like protein 3 |
| P61626 | 24.32 | 24 | 4 | 125.746 | 9.551 | *2.709* | 15.453 | Lysozyme C |
| P02656 | 34.34 | 4 | 3 | 15.151 | 9.381 | 8.698 | 5.949 | Apolipoprotein C-III |
| P02655 | 17.82 | 2 | 2 | 10.761 | 9.099 | 9.229 | 5.023 | Apolipoprotein C-II |
| P05164 | 36.64 | 103 | 26 | 522.249 | 8.271 | *3.049* | 14.035 | Myeloperoxidase |
| Q58FG1 | 8.85 | 27 | 4 | 148.865 | 7.149 | 5.468 | 10.247 | Putative heat shock protein HSP 90-alpha A4 |
| Q99856 | 13.66 | 12 | 8 | 64.485 | 6.936 | 8.118 | 6.186 | AT-rich interactive domain-containing protein 3A |
| F6SBX2 | 1.81 | 2 | 2 | 145.973 | 6.568 | *4.312* | 10.164 | Isoleucine-tRNA ligase |
| O00425 | 11.05 | 15 | 9 | 92.208 | 6.395 | 6.444 | 5.990 | Insulin-like growth factor 2 mRNA-binding protein 3 |
| Q96KK5 | 38.28 | 79 | 5 | 462.303 | 6.321 | 7.461 | 6.062 | Histone H2A type 1-H |
| P02768 | 46.14 | 199 | 33 | 965.068 | 6.175 | 6.800 | *4.290* | Serum albumin |
| Q9Y3Z3 | 5.91 | 4 | 4 | 46.783 | 6.080 | *2.390* | 5.689 | SAM domain and HD domain-containing protein 1 |
| A6NDF1 | 10.09 | 2 | 2 | 13.262 | 5.907 | 8.791 | 7.723 | Putative uncharacterized protein C10orf58 |
| Q15651 | 15.15 | 5 | 3 | 23.368 | 5.643 | *3.779* | *4.847* | High mobility group nucleosome-binding domain-containing protein 3 |
| F5H2E5 | 2.94 | 2 | 2 | 17.092 | 5.588 | *4.805* | *4.661* | Pyrin |
| Q3ZCW2 | 11.05 | 2 | 2 | 10.446 | 5.507 | *2.530* | *3.334* | Galectin-related protein |
| P29966 | 9.04 | 4 | 2 | 18.870 | 5.418 | *2.860* | 8.018 | Myristoylated alanine-rich C-kinase substrate |
| P01009 | 13.64 | 9 | 7 | 48.002 | 5.288 | 5.064 | *4.007* | Alpha-1-antitrypsin |
| P01859 | 8.28 | 4 | 3 | 25.896 | 5.230 | 5.550 | *4.400* | Ig gamma-2 chain C region |
| P0CG05 | 30.19 | 3 | 3 | 14.669 | 5.147 | *4.990* | *3.616* | Ig lambda-2 chain C regions |
| Q9UL25 | 11.56 | 2 | 2 | 41.816 | *4.544* | *2.347* | 6.500 | Ras-related protein Rab-21 |
| P68431 | 33.82 | 206 | 8 | 1325.565 | *4.471* | 5.001 | *4.707* | Histone H3.1 |
| Q13228 | 16.53 | 12 | 8 | 56.722 | *4.451* | 5.148 | 7.540 | Selenium-binding protein 1 |
| E9PEW5 | 1.07 | 3 | 2 | 40.191 | *4.404* | *2.655* | 11.184 | Myosin X |
| P80723 | 45.81 | 14 | 8 | 69.884 | *3.095* | *1.117* | 5.199 | Brain acid soluble protein 1 |
| F5H8B6 | 12.56 | 4 | 3 | 31.042 | *3.017* | *1.905* | 6.782 | Protinase 3 |
| P59665 | 19.15 | 5 | 2 | 19.143 | *2.493* | *2.758* | 6.337 | Neutrophil defensin 1 |
| D9YZU8 | 81.63 | 410 | 12 | 2322.160 | *1.193* | 5.930 | 8.381 | Hemoglobin, gamma A |
