# Supplementary material for: Qualitative and Quantitative Comparison of the Proteome of Erythroid Cells Differentiated from Human iPSCs and Adult Erythroid Cells by Multiplex TMT Labelling and NanoLC-MS/MS
Source: PLoS One. 2014 Jul 14;9(7):e100874. doi: 10.1371/journal.pone.0100874 (PMC4096399; doi:10.1371/journal.pone.0100874)
Supplement: Table S6 — Comparison of the level of histone proteins between erythroid cells differentiated from adult peripheral blood (PB) compared to C19, OCE1 and OPM2 CD34+ cells, and between cord blood (CB) compared to C19, OCE1 and OPM2 CD34+ cells, at day 8 in culture. (DOCX) [file pone.0100874.s010.docx]

**Table S6. Level of histone proteins in erythroid cells differentiated from iPSCs C19, OCE1 and OPM2 compared to from PB progenitors.**

| **Accession** | **Peptides** | **C19/PB** | **C19/CB** | **OCE/PB** | **OCE/CB** | **OPM/PB** | **OPM/CB** | **Description** |
| --- | --- | --- | --- | --- | --- | --- | --- | --- |
| P16403 | 17 | 2.714 | 2.792 | 3.047 | 3.194 | 2.364 | 2.436 | Histone H1.2 |
| P16402 | 20 | 1.796 | 1.718 | 2.248 | 2.121 | 1.6 | 1.538 | Histone H1.3 |
| P10412 | 18 | 2.718 | 2.544 | 2.862 | 2.694 | 2.479 | 2.309 | Histone H1.4 |
| P16401 | 17 | 2.905 | 2.778 | 2.687 | 2.68 | 2.559 | 2.439 | Histone H1.5 |
| Q92522 | 13 | 1.892 | 2.187 | 1.977 | 2.299 | 1.431 | 1.689 | Histone H1x |
| Q96KK5 | 5 | 6.321 | 5.288 | 7.461 | 6.242 | 6.062 | 5.071 | Histone H2A type 1-H |
| Q8IUE6 | 3 | 3.882 | 3.726 | 3.028 | 2.906 | 3.639 | 3.492 | Histone H2A type 2-B |
| Q16777 | 5 | 18.435 | 10.477 | 20.578 | 11.695 | 14.334 | 8.146 | Histone H2A type 2-C |
| P16104 | 5 | 4.35 | 4.234 | 4.247 | 4.134 | 2.755 | 2.733 | Histone H2A.x |
| P0C0S5 | 4 | 2.688 | 2.789 | 2.354 | 2.519 | 2.417 | 2.581 | Histone H2A.Z |
| Q8N257 | 8 | 2.583 | 3.14 | 2.538 | 3.085 | 1.57 | 1.908 | Histone H2B type 3-B |
| P68431 | 8 | 4.471 | 3.741 | 5.001 | 4.313 | 4.707 | 3.864 | Histone H3.1 |
| P84243 | 8 | 2.521 | 2.816 | 2.628 | 2.951 | 2.345 | 2.62 | Histone H3.3 |
| P62805 | 6 | 4.993 | 4.885 | 4.874 | 4.723 | 4.421 | 4.28 | Histone H4 |
| O75367 | 6 | 2.717 | 3.165 | 1.765 | 2.038 | 2.717 | 3.234 | Core histone macro-H2A.1 |
| F5H022 | 6 | 4.651 | 4.524 | 3.878 | 3.914 | 2.818 | 2.82 | Histone H1F0 |
